# Health-related quality of life in family caregivers of autistic adults

**DOI:** 10.3389/fpsyt.2023.1290407

**Published:** 2023-12-18

**Authors:** Sophia Dückert, Sabine Bart, Petia Gewohn, Hannah König, Daniel Schöttle, Alexander Konnopka, Pascal Rahlff, Frank Erik, Kai Vogeley, Holger Schulz, Nicole David, Judith Peth

**Affiliations:** ^1^Department of Medical Psychology, University Medical Center Hamburg-Eppendorf, Hamburg, Germany; ^2^Department Health Sciences, Faculty Life Sciences, Hamburg University of Applied Sciences, Hamburg, Germany; ^3^Department of Psychiatry and Psychotherapy, University Medical Center Hamburg-Eppendorf, Hamburg, Germany; ^4^Department of Health Economics and Health Services Research, University Medical Center Hamburg-Eppendorf, Hamburg, Germany; ^5^Department of Psychiatry, Faculty of Medicine and University Hospital Cologne, University of Cologne, Cologne, Germany

**Keywords:** autism spectrum disorder, adults, family caregivers, quality of life, mental wellbeing, caregiver burden

## Abstract

**Introduction:**

Family members of autistic individuals often provide support for their autistic relative throughout the lifespan which can lead to massive burden themselves. Reduced health-related Quality of Life (HRQoL) in family caregivers is assumed; however, only a handful studies on the HRQoL of family caregivers providing care to adult relatives exist as opposed to autistic children. Thus, the current study aimed to (i) investigate the current state of physical and mental HRQoL of family caregivers of autistic adults compared to the general population, and (ii) examine caregiver-related (e.g., age, subjective caregiver burden) and care recipient-related variables (e.g., symptom severity, utilization of formal services) explaining variance in the caregivers’ HRQoL.

**Methods:**

*N* = 149 family caregivers completed a nationwide online survey, including the Short-Form Health Survey (SF-8) in order to assess the HRQoL. T-tests were used to compare the HRQoL of family caregivers with the general population. Bivariate correlational and multiple linear regression analyses were conducted in order to identify predictors explaining variance in family caregivers’ HRQoL.

**Results:**

Family caregivers of autistic adults reported significantly lower physical (*M* = 46.71, *SD* = 8.72, Cohen’s *d* = 0.42) and mental HRQoL (*M* = 40.15, *SD* = 11.28, Cohen’s *d* = 1.35) compared to the general population. Multiple linear regression with the mental HRQoL as the outcome showed a significant model (*F*(11, 95) = 5.53, *p* < .001, adj. *R*^2^ = .32) with increased subjective burden explaining most of the variance in mental HRQoL (*ß* = .32, GDW = .141, *p* < .001). Multiple linear regression analysis with the outcome physical HRQoL did not reveal a statistically significant model (*F*(11,95) = 1.09, *p* = .38). However, bivariate analyses also showed a positive correlation with the subjective caregiver burden (*r*= .20, *p* < .05).

**Discussion:**

Findings highlight the need to consider HRQoL (and caregiver burden) of family caregivers of autistic adults in several healthcare settings to monitor a potential comprised health status in early stages, with the long-term goal to improve family caregivers’ HRQoL.

## 1 Introduction

Core symptoms for the diagnosis of Autism Spectrum Disorder (ASD) include persistent specifics in social communication/interaction and restrictive, repetitive, and inflexible behavior patterns (1). In addition, somatic and mental comorbidities and challenging behaviors (e.g., aggression, self-injuring, and attempting suicide) are common (2, 3). One-third to one-half of autistic individuals have an accompanying intellectual disability (ID) (4, 5). In ASD, these specifics are very heterogeneous but often result in the need for support/assistance/care (for purpose of simplification, mentioned as “care” below). Typical caregiving demands include assistance with housekeeping, transportation, personal care (e.g., dressing, eating, toileting) (6) as well as distinct challenges, such as the need for mediation in social interactions, inflexible daily routines, or inappropriate behaviors (7, 8).

Usually, family caregivers are the most important source of support for autistic individuals (7). Accordingly, “diagnosis of autism affects not just the individual, but the entire family” [p. 45, (9)]. Especially when transitioning into adulthood and massive barriers impede adequate healthcare for autistic adults (e.g., pediatric services are no longer available, lack of funding, long waiting lists for diagnostics/treatment), autistic adults rely on the support of family caregivers [e.g., (grand) parents, partners/spouses, siblings, adult children] (9–11). Although caring for a loved one with autism also include positive effects on family caregivers (12), caregiving is typically time-intensive and exhausting (13). As autistic relatives enter adulthood, the duration and ongoing demands of caregiving can accumulate and lead to increased stress for family caregivers (14, 15), and have several negative effects on their lives, as shown by increased subjective caregiver burden (16, 17). Many family caregivers of autistic adults struggle with the care-associated responsibilities, while balancing everyday life, social interactions, and occupational responsibilities (15, 18–20). Loss of employment and high costs associated with care were discussed to lead to major financial burden (15, 20). Family caregivers of autistic adults also seem to be experiencing increased physical and mental health problems (21–23). For example, this population reported significantly higher emotional distress compared to caregivers of patients with schizophrenia and are at higher risk to develop health problems themselves (24).

Furthermore, compromised levels of health-related Quality of Life (HRQoL) in autistic adults’ family caregivers were reported (25, 26), which refers to “how well a person functions in their life and his or her perceived wellbeing in physical, mental, and social domains of health” (27). HRQoL captures information on both physical and mental health status and its impact on Quality of Life (QoL). It is a useful indicator of overall health and is suitable for prevention and early detection of physical or mental diseases (28–32). As a so-called “patient-reported outcome,” HRQoL comprises the perspective of patients, making it suitable for validating healthcare services and interventions and ensuring the provision of high-quality services (33). However, evidence on HRQoL in family caregivers of autistic adults is lacking, with only a handful of international studies on this topic (25, 26, 34–36), and no data from Europe. Still, existing evidence indicates that HRQoL of family caregivers of autistic adults is reduced – without HRQoL scores having been compared with other populations yet.

In attempt to identify the underlying mechanisms of this (presumed) finding, a number of potential predictors have been investigated. For instance, a systematic review by Sonido et al. (37) (*N* = 23 included studies) scrutinized potential predictors of mental wellbeing of family caregivers of autistic adults. Due to the limited amount of available evidence, a broad variety of outcomes were considered (e.g., psychological stress, mental wellbeing, HRQoL, QoL), without categorizing them as discrete components to provide discriminant evidence. In addition, the picture on several potential predictors is less clear-cut. Discrepant or lacking evidence has been identified for various caregiver-related and care recipient-related predictors: Regarding caregiver-related, and especially sociodemographic predictors, two studies found older caregivers having better mental wellbeing (26, 38), while other studies could not support this finding (39, 40). Furthermore, Grootscholten et al. (24) reported that parental and spousal caregivers show higher psychological burden (i.e., stress, depression, anxiety) compared to other family caregivers (e.g., siblings, children). With respect to school education, Greenberg et al. (40) found higher educated mothers to experience better psychological wellbeing, whereas other studies did not support such finding (22, 26, 38). In addition, predictors directly related to caregiving demands were examined: The higher the subjective caregiver burden, the lower the parental HRQoL (41). However, no significant associations were found between parental financial burden and actual time spent due to caregiving demands and HRQoL (26, 35).

Besides caregiver-related predictors, different care recipient-related predictors were assumed to be associated with family caregivers’ wellbeing, focusing on clinical and healthcare utilization outcomes. Rattaz et al. (22) found that more severe ASD symptoms in care recipients predicted lower caregivers’ global QoL. This prediction was not confirmed in other studies (21, 26) or only between severity of behavioral symptoms and parental HRQoL (42). Contradictory results were also found with respect to the presence of care recipients’ comorbid ID, with one study showing improved caregiver mental HRQoL when the care recipient has an ID (26), while other studies did not (39, 43). No significant associations were found between utilization of formal services by the care recipient and mental wellbeing outcomes in caregivers (26, 39). Moreover, in parents of autistic children, depressive symptoms increased when the child was diagnosed with ASD at a higher age (44). However, age at ASD diagnosis as a potential predictor of HRQoL and in caregivers of autistic adults has not been examined yet.

These previous published studies provide important evidence but reveal some limitations. First, the observed primary outcome differed highly over studies (e.g., psychological distress, mental-wellbeing, QoL, HRQoL). HRQoL was found to be a valuable outcome in various healthcare settings, but most predictors were investigated only in the context of the aforementioned related constructs. Moreover, when considering HRQoL, also very little is known about the distinction between mental and physical HRQoL. Second, the picture for several potential predictors is less clear. Inconsistent or lacking evidence has been identified for various caregiver-related and care recipient-related variables. Third, most studies included mainly parental caregivers and did not investigated, for example, partners/spouses and siblings (39, 42). Moreover, as comparisons to reference populations (e.g., non-clinical populations, family caregivers of relatives with other chronic diseases) are lacking, existing evidence cannot be interpreted properly.

Thus, the present study aimed for the first time to (i) investigate the current state of physical and mental HRQoL in family caregivers of autistic adults compared to the general population in Germany, and (ii) analyze a comprehensive set of previously reported caregiver-related and care recipient-related predictors of both physical and mental HRQoL in family caregivers of autistic adults. To our knowledge, this is the first broad investigation of physical and mental HRQoL in family caregivers of autistic adults. Results from this study might contribute important information by identifying parameters associated with impaired HRQoL in family caregivers and, consequently, providing an indication of the need for action within professional healthcare settings, with the long-term goal of improving the HRQoL in this underserved population, as called for by the scientific community (9, 37, 45).

## 2 Methods

This cross-sectional observational study was conducted as part of the research project *BarrierfreeASD* (46). Ethical approval was obtained from the Local Psychological Ethics Committee at the Center for Psychosocial Medicine at the University Medical Center Hamburg-Eppendorf (#LPEK-0227; Dec. 2020), and the study was conducted in accordance with the Declaration of Helsinki. Participation was voluntary and anonymous and informed consent was obtained before participation. An inconvenience allowance was not paid. The *BarrierfreeASD* project has been preregistered with the Open Science Framework.^1^ Two autistic researchers were part of the *BarrierfreeASD* project and were involved in developing the online survey, the data collection and the interpretation of the results. Furthermore, research was conducted in close collaboration with the study’s collaborative network, including autism-related associations and family caregiver-related associations, which guided the research process throughout. This manuscript was conducted in accordance with the Journal Article Reporting Standards for Quantitative Research in Psychology (JARS) (47).

### 2.1 Participants

This study analyzed data from *N* = 149 family caregivers of autistic adults. Participants were recruited throughout Germany using purposive, quota and snowball sampling methods via the study’s network of collaborating partners, publicly available contacts from autism-related associations (including self-help and caregiver groups), and social media. Therefore, response rates could not be calculated. Participants were included if they were at least 18 years old, a first- or second-degree relative or partner/spouse of an autistic adult (care-recipient), and had sufficient language skills. The nationwide online survey was distributed using LimeSurvey (48). The data collected included questions on sociodemographic and clinical information about the family caregiver, details about the autist adult, and details about the informal care provided (see measurement section for details). Sample characteristics of caregivers and care recipients are presented in Table 1.

**Table 1:** Sample characteristics (*N* = 149).

	*N*	*n* (%) / *M (SD)*
Family caregiver-related characteristics		
Age (in years)	149	51.95 (9.48) Min-Max: 20–65
Sex	149	
Female		130 (87.2)
Male		18 (12.1)
Diverse		1 (0.7)
Relationship to autistic adult	140	
Parent		99 (70.7)
Partner/spouse		19 (13.6)
Child		14 (10)
Sibling		4 (2.9)
Other		4 (2.9)
Marital Status	149	
Married/Relationship		116 (77.9)
Divorced		17 (11.4)
Single		9 (6)
Widowed		4 (2.7)
Married (in separation)		3 (2)
Highest school education	147	
University entrance qualification (A-Levels)		110 (74.8)
Secondary school certificate		30 (20.4)
First school certificate		7 (4.8)
Highest professional degree	145	
University degree		82 (56.6)
Vocational school		36 (24.8)
Technician/master school		18 (12.4)
No professional degree		4 (2.8)
Engineering school		2 (1.4)
Other		3 (2.1)
Employment status^a^	145	
Part-time employed		64 (44.1)
Full-time employed		40 (27.6)
Not employed		20 (13.8)
Minor employment (Minijob)		8 (5.5)
In vocational training		2 (1.4)
Not applicable		12 (8.3)
Care recipient-related characteristics		
Age (in years)	140	26.69 (9.75) Min-Max: 18–60
Sex	140	
Female		32 (22.9)
Male		103 (73.6)
Diverse		5 (3.6)
Diagnosis	139	
Asperger Syndrome		96 (69.1)
Childhood Autism		19 (13.7)
Atypical Autism		18 (12.9)
Other		6 (4.3)
Co-occuring ID (yes)	140	13 (9.3)

N, sample size; SD, standard deviation; M, mean; ID, intellectual disability. ^a^ multiple responses were possible.

### 2.2 Measurement

#### 2.2.1 Caregiver-related measurements

*Sociodemographic data* about family caregivers included age, sex, marital status, relationship to an autistic relative, school education, and employment status. *Treatment-related expenses* were measured using the questionnaire of Mory et al. (49) which assesses treatment-related expenses (e.g., medication, co-payments for therapies), practical living support (e.g., rent, help in the household), and extraordinary expenses (e.g., debt repayments, special purchases) for the autistic relative in the past year (sum in Euro). *Subjective caregiver burden* was measured using the CarerQol-7D as a feasible, valid and reliable instrument (50, 51). Seven dimensions were included in the CarerQol-7D: fulfillment, relational, mental health, social, financial, perceived support, and physical dimension. Each item measured one dimension and was rated with “no,” “some,” or “a lot.” Tariff-based sum scores were calculated, ranging from 0 (worst informal care situation) to 100 (best informal care situation) (52). *Objective caregiver burden.* Self-developed items were used to measure the capacity of informal care. Participants were asked to indicate whether they had provided informal care for the adult relative with autism in the past 6 months. If they agreed, the number (in days) and the average duration per appointment (in hours) of each type of informal care (household, personal care, intake of medication, visits to administrative authorities, doctor’s appointments, finances, other) were estimated. For the purposes of this study, the total time for informal care (in hours) was calculated.

#### 2.2.2 Care recipient-related measurements

*Clinical information* on the care recipient comprised the age at ASD diagnosis and the presence of an ID. As there was no standardized proxy measurement to assess the care-recipients’ symptom severity of ASD, family caregivers rated their relatives’ symptom severity based on two self-developed items analogous to the “Diagnostic and Statistical Manual of Mental Disorders” (DSM-5) (1) classification of severity/required support for the two main diagnostic criteria (A. Persistent deficits in social communication and social interaction, B. Restricted, repetitive patterns of behavior, interests, or activities). Family caregivers rated the following two items on a 5-point Likert scale (“1 = none” to “5 = severe”): “How much does your autistic relative require support due to difficulties in interpersonal communication and social interactions?” and “How much does your autistic relative require support because she/he is holding on to behavioral habits, routines, or interests (e.g., difficulties in self-organization or dealing with change)?” Ratings for the two domains were used as separate independent variables for the analyses. Number of received professional healthcare and support services in the past 6 month were collected using modified versions of the German Questionnaire for the Assessment of Health Services in Old Age (FIMA) (53) and the Questionnaire on the Utilization of Medical and Nonmedical Care Services in Mental Disorders (FIMPsy) (54).

#### 2.2.3 Health-related quality of life (outcome)

*Health-related Quality of Life (HRQoL)* was measured using the Short-Form Health Survey (SF-8) because it is a generic instrument to assess HRQoL in physically and mentally health as well as in burdened populations (55). As a parsimonious and user-friendly instrument, each of the eight single-items assessed one dimension of the longer SF-36 health survey and allowed the calculation of a physical component scale (PCS) and a mental component scale (MCS) (56). Items were scored on a 5-point Likert scale and the PCS and MCS were derived using an algorithmic norm-based scoring procedure, with higher scores indicating better HRQoL (0–100) (55). The SF(−8/−12/−36) is used both nationally and internationally, which allows comparisons between different populations. Previous research has shown strong reliability (parallel test reliability *r* = 0.82) and validity of the MCS (55).

### 2.3 Data analysis

Data were analyzed using IBM SPSS version 27 (57). Missing data were not imputed. All decisions regarding the statistical significance of findings were made using a criterion alpha level of 0.05.

Three distinct analyses were performed. First, both physical and mental HRQoL scores (PCS and MCS of the SF-8) were compared with those of the general population in Germany (*N* = 2,552) using two two-tailed t-tests for independent samples (58). Normative data were taken from a study by Beierlein et al. (59). Interpretation of effect size (Cohen’s d) were based on the conventions of Cohen (60) (small effect size: *d* = 0.2, medium effect size: *d* = 0.5, large effect size: *d* = 0.8).

Second, bivariate correlational analyses (Pearson’s correlation for metric variables, point-biserial correlations for one dichotomous and one metric variable, and Phi coefficients for both dichotomous variables) were performed in order to examine correlations between physical and mental HRQoL and variables. Interpretation of effect sizes (*r*) were based on the conventions of Cohen (60) (small effect size: *r* = 0.10, medium effect size: *r* = 0.30, large effect size: *r* = 0.50).

Third, two multiple linear regression models (PCS and the MCS of the SF-8 as dependent variables) were calculated to examine potential predictors of the family caregivers’ physical and mental HRQoL as a comprehensive set of predictors. An *a priori* power calculation revealed a required sample size of *N* = 87, assuming a moderate to high effect of *R*^2^ = 0.18, based on effect sizes of previous studies, with a statistical power of 0.80 and an alpha level of 0.05 for 11 predictors (61). Multicollinearity between predictors was assessed using the variance inflation factor [VIF; critical VIF > 2.5 (62)]. All other assumptions of multiple regression analysis could also be verified (linearity, normality, homoscedasticity, independence of errors). Predictors were entered into regression analyses in continuous or binary categorical data format in one block. To be included in regression analyses, categorical variables with more than two values/categories were recoded into dichotomous format, e.g., relationship to care recipient (1 = parental caregiver, 0 = non-parental caregiver), education (1 = A-levels/tertiary school education, 0 = no A-levels), ID (1 = ID, 0 = no ID, see Table 2). Interpretations of effects sizes (*R*^2^) were based on the recommendations of Cohen (60) (small effect size: *R*^2^ = 0.01, medium effect size: *R*^2^ = 0.09, large effect size: *R*^2^ = 0.25). In order to compare the relative importance of predictors, dominance analysis was performed using R version 3.6.2 (63) package “yhat” (64). The General Dominance Weights (GDW) of predictors were calculated by averaging the squared semipartial correlations across all of the possible subset models. This measure indexes a variable’s contribution to the prediction of the dependent variable, by itself and in combination with the other predictors (65).

**Table 2:** Descriptive statistics of variables.

Variable	*N*	*M* (*SD*)	Min. – Max.	*n* (%)
Dependent variables				
Physical HRQoL (PCS of SF-8)	120	46.71 (8.72)	25.9–61.8	
Mental HRQoL (MCS of SF-8)	120	40.15 (11.28)	15.08–62.86	
Independent variables				
Caregiver-related variables				
Age (years)	149	51.95 (9.48)	20–65	
Relationship (parental)	149			99 (70.7)
School education (A-Levels)	147			110 (74.8)
Treatment related expenses (Euro)	125	4968.82 (7490.66)	0–49,900	
CarerQol-7D (sum score)	114	56.08 (14.94)	11–89	
Informal care (hours)	125	1540.54 (3163.73)	0–23,280	
Care recipient-related variables				
Severity (Communication)	129	3.74 (0.99)	1–5	
Severity (Behavior)	129	3.77 (1.01)	1–5	
Age at diagnosis (years)	140	17.44 (12.02)	3–57	
ID (yes)	140			13 (9.3)
Formal services (sum score)	139	1.21 (1.16)	0–5	

N, sample size; M, mean; SD, Standard Deviation; HRQoL, health-related Quality of Life; SF-8, Short Form Health-Survey; PCS, Physical Component Scale; MCS, Mental Component Scale; ID, Intellectual Disability.

## 3 Results

Descriptive statistics of the dependent and independent variables are shown in Table 2.

### 3.1 Comparison of HRQoL with general population

**Figure 1:**
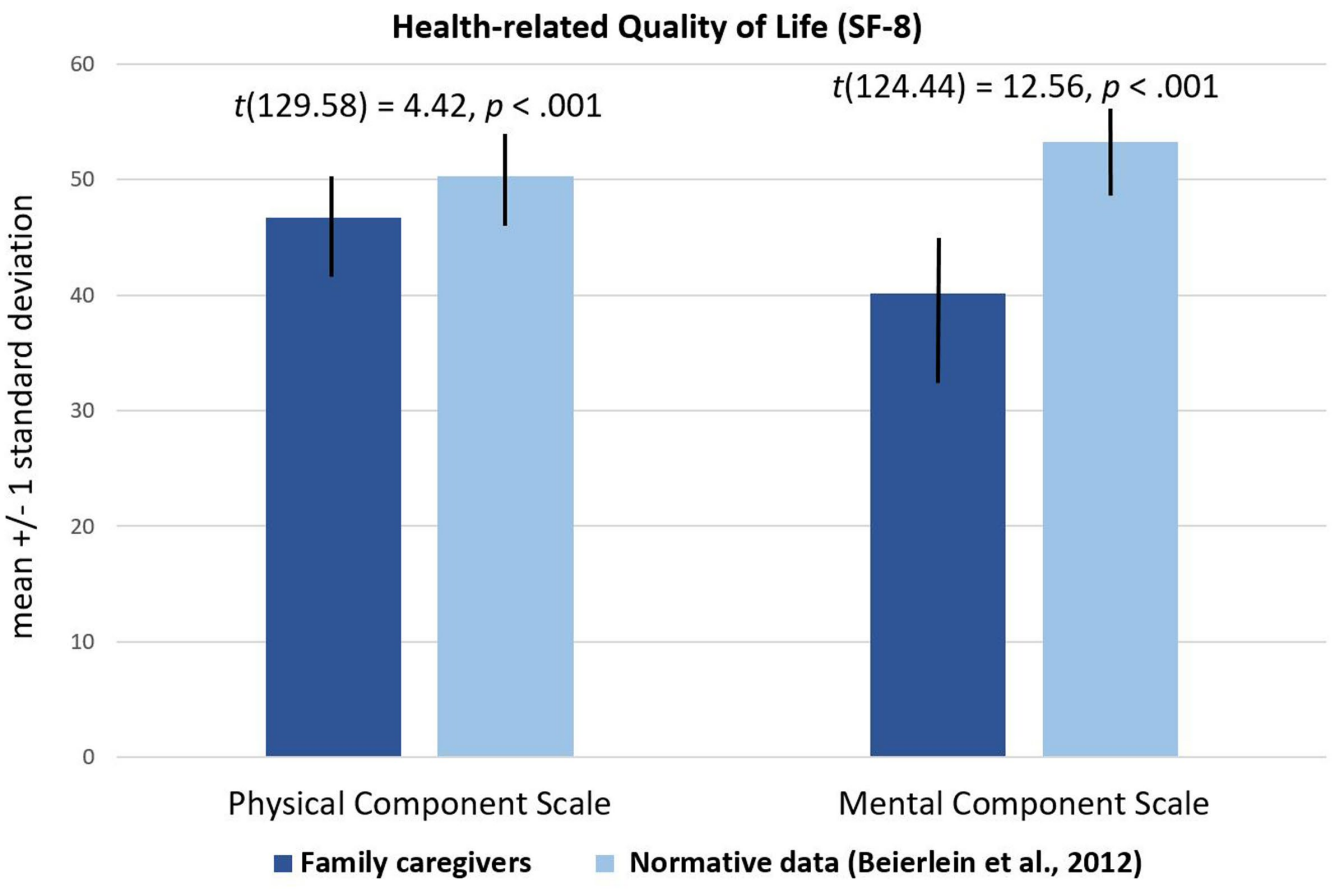
Comparison of HRQoL in family caregivers of autistic adults and in the general population.

*Physical HRQoL.* Data of the study (*N* = 120) showed a mean PCS score of *M* = 46.71 (*SD* = 8.72; see Figure 1). The German normative sample (*N* = 2,552) (59) rated a mean PCS score of *M* = 50.3 (*SD* = 8.39,). T-test for independent samples indicated that the difference between the two samples was statistically significant for the PCS [*t*(129.58) = 4.42, *p* < 0.001, Cohen’s *d* = 0.42], indicating a significantly lower physical HRQoL for caregivers of autistic adults compared to the general population with a small effect (60).

*Mental HRQoL.* Data of the study (*N* = 120) showed a mean MCS score of *M* = 40.15 (*SD* = 11.28; see Figure 1). Normative data from Germany (59) reported for the general population (*N* = 2,552) a mean MCS score of *M* = 53.23 (*SD* = 7.82). T-test for independent samples revealed statistically significant difference for MCS scores [*t*(124.44) = 12.56; *p* < 0.001, Cohen’s *d* = 1.35], indicating a significantly lower mental HRQoL for caregivers of autistic adults compared to the general population with a large effect (60).

### 3.2 Correlation analyses

Bivariate analyses found several significant weak to high correlations between included HRQoL and predictors (see Table 3 for details) (60). *Physical HRQoL* was significantly positively correlated with the subjective caregiver burden (*r* = 0.20, *p* = 0.031) and negatively correlated with the symptom severity regarding behavior symptoms (*r* = −0.21, *p* = 0.024). *Mental HRQoL* was positively correlated with caregiver’s age (*r* = 0.19, *p* = 0.035), subjective caregiver burden (*r* = 0.44, *p* < 0.001), care recipient’s comorbid ID (*r* = 0.18, *p* = 0.048), and the amount of received formal services (*r* = 0.21, *p* = 0.005). Inverse correlations were found with the school education (*r* = −0.27, *p* = 0.003) and the symptom severity regarding behavior symptoms (*r* = −0.27, *p* = 0.005). Remaining variables did not show significant correlations with the physical and mental HRQoL.

**Table 3:** Correlation matrix (*N* = 120).

	1	2	3	4	5	6	7	8	9	10	11	12
1. Caregiver’s age (years)												
2. Relationship (parental)	0.45**											
3. School education (A-Level)	−0.16	−0.22*										
4. Treatment-related expenses (Euro)	0.04	−0.02	0.16									
5. CarerQol-7D (sum score)	0.05	0.00	−0.27**	−0.10								
6. Informal care (hours)	−0.03	0.11	−0.12	0.18	0.01							
7. Severity (social communication)	0.06	0.11	−0.05	0.09	−0.05	0.33**						
8. Severity (behavior)	0.02	0.12	−0.16	0.02	−0.20*	0.18	0.59**					
9. Age at ASD diagnosis (years)	−0.17*	−0.48**	0.06	−0.03	0.08	−0.21*	−0.11	−0.12				
10. ID (yes)	−0.11	0.10	0.01	−0.05	0.08	0.20*	0.22*	0.15	−0.15			
11. Formal services (sum score)	0.03	−0.04	0.10	0.22*	−0.11	0.01	0.27**	0.14	0.07	−0.16		
12. PCS (SF-8)	−0.12	−0.12	0.04	−0.05	0.20*	−0.11	−0.13	−0.21*	0.10	0.04	−0.08	
13. MCS (SF-8)	0.19*	0.14	−0.27**	−0.08	0.44**	0.01	−0.13	−0.27**	−0.13	0.18*	−0.26**	0.21*

PCS, Physical Component Scale; MCS, Mental Component Scale; SF-8, Short Form Health Survey; ID, Intellectual Disability. *p < 0.05; **p < 0.01. Bold indicates significant values.

### 3.3 Predictors of HRQoL

Multiple linear regression analysis with the outcome PCS scores did not reveal a statistically significant model [*F*(11, 95) = 1.09, *p* = 0.38]. For details, see Table 4.

**Table 4:** Multiple linear regression analyses (*N* = 107).

	Physical HRQoL				Mental HRQoL					
Predictor	*b*	*SE*	*ß*	95% *CI*	*b*	*SE*	*ß*	95% *CI*	GDW	VIF
Caregiver										
Age (years)	−0.17	0.11	−0.84	[−0.40, 0.05]	0.20	0.12	0.16	[−0.05, 0.44]	0.018	1.53
Relationship (parental)	0.40	2.74	0.02	[−5.05, 5.85]	−3.00	2.98	−0.12	[−8.92, 2.92]	0.006	2.07
School education (A-Level)	−0.02	2.29	−0.01	[−4.56, 4.52]	−5.78*	**2.48**	**−0.22**	**[−10.71, −0.85]**	**0.068**	1.33
Treatment-related expenses (Euro)	<0 0.001	<0 0.001	0.02	[0.00, 0.00]	<0 0.001	<0 0.001	0.01	[< 0.001, <0.001]	0.003	1.14
CarerQol-7D (sum score)	0.11	0.06	0.19	[−0.01, 0.23]	0.24***	**0.01**	**0.32**	**[0.11, 0.37]**	**0.141**	1.23
Informal care (hours)	0.00	0.00	−0.08	[−0.01, 0.00]	<0.001	<0.001	−0.03	[−0.01, 0.01]	<0.001	1.21
Care recipient										
Severity (social communication)	−0.57	1.14	−0.06	[−2.83, 1.70]	−0.49	1.24	−0.04	[−2.95, 1.97]	0.013	1.74
Severity (behavior)	−0.52	1.11	−0.06	[−2.73, 1.69]	−2.05	1.21	−0.18	[−4.45, 0.35]	0.039	1.65
Age at diagnosis (years)	0.07	0.09	0.09	[−0.12, 0.25]	−0.20*	**0.10**	**−0.20**	**[−0.40, −0.01]**	**0.023**	1.56
ID (yes)	1.41	2.88	0.05	[−4.31, 7.13]	4.94	3.13	0.14	[−1.28, 11.15]	0.025	1.18
Formal services (sum score)	−0.29	0.77	−0.04	[−1.81, 1.24]	−1.77*	**0.84**	**−0.19**	**[−3.42, −0.11]**	**0.054**	1.22

Outcomes: physical and mental HRQoL (PCS and MCS of SF-8). Model fit (physical HRQoL): F(11, 95) = 1.09, *p* = 0.38. Model fit (mental HRQoL): F(11, 95) = 5.53, *p* < 0.001, R^2^ = 0.32. ID, intellectual disability; SE, Standard Error; CI, Confidence Interval; GDW, General Dominance Weights; VIF, Variance Inflation Factor. *p < 0.05; **p < 0.01; ***p < 0.001. Bold indicates significant values.

Multiple linear regression with the mental HRQoL as the outcome showed a significant model [*F*(11, 95) = 5.53, *p* < 0.001], with an adjusted *R*^2^ of 0.32 (see Table 4). Significant results were obtained for the following caregiver variables: school education (*ß* = −0.22, GDW = 0.068, *p* < 0.05) and CarerQol-7D sum score (*ß* = 0.32, GDW = 0.141, *p* < 0.001). Higher school education and lower CarerQol-7D sum scores predicted lower MCS scores. For care recipient variables, the age at diagnosis (*ß* = −0.20, GDW = 0.023, *p* < 0.05) and the number of formal services (*ß* = −0.19, GDW = 0.054, *p* < 0.05) were significant predictors: high age at diagnosis and a higher number of formal services used by the autistic adult predicted lower mental HRQoL. Remaining predictors were not significant.

## 4 Discussion

To our knowledge, the present study is the first to provide an assessment of physical and mental HRQoL in family caregivers of autistic adults in Germany. The main results show that both mental and physical HRQoL were significantly reduced compared to the German normative population. Especially family caregivers’ mental HRQoL was considerably lower than that of the normative population. To shed light on the reduced HRQoL, bivariate analyses was conducted in order to detect correlations between the physical and mental HRQoL and the variables: Subjective caregiver burden and ASD severity in terms of repetitive and restrictive behaviors, interests, and activities correlated with both physical and mental HRQoL. Furthermore, a comprehensive set of potential predictors of HRQoL was examined: The set of variables investigated in this study showed that the strongest predictor of mental HRQoL was the subjective caregiver burden. Understanding the underlying causes of reduced HRQoL in family caregivers of autistic adults might help to inform professionals in the healthcare system about especially vulnerable individuals and to develop and decide about adequate support strategies.

### 4.1 Reduced HRQoL in family caregivers of autistic adults

In contrast to previous studies, the reported HRQoL scores were compared to the general population to provide a reference. Here, family caregivers of autistic adults reported significantly lower physical and mental HRQoL compared to the German general population. The HRQoL scores found in the current study are comparable to a study from the United States that investigated HRQoL in parental caregivers of young autistic adults (25), suggesting a ubiquitously reduced HRQoL in caregivers of autistic adults regardless of country of origin. However, the mental HRQoL scores in our study were slightly lower than the findings of Lee and Shivers (25). In addition, compared to family caregivers of patients with severe mental illness (66) or Down Syndrome (67), participants in the current study reported the lowest mental HRQoL scores. It is questionable whether, for example, autism-specific care needs or differences in healthcare structures due to regional disparities or inequalities in the healthcare of autistic adults and their family caregivers compared to other mental or physical disorders lead to these findings. As poor HRQoL scores are associated with several negative outcomes in the general population such as multimorbidity (68) or higher mortality risk (69), these findings are a matter of great concern and should motivate the development of support systems. Furthermore, the findings emphasize the importance of HRQoL as a relevant outcome not only in autistic adults but also in their family caregivers in order to obtain early indications of the family caregivers’ health status (70). Professionals working with autistic adults and their families should be aware that provided care may have an impact on the physical and mental health status (41). In addition, the findings highlight the need to address the lack of evidence on physical HRQoL in future research.

### 4.2 Bivariate and multiple regression analyses of family caregivers’ HRQoL

The results of this study revealed caregiver-related and care recipient-related predictors explaining variance of HRQoL in family caregivers of autistic adults, which were predominantly differing between the two outcomes physical and mental HRQoL. However, two variables showed significant correlations with both physical and mental HRQoL in the bivariate analyses: Subjective caregiver burden and ASD severity regarding repetitive and restrictive behaviors, interests, and activities. Also in the multiple regression model with the *mental HRQoL* as dependent variable, subjective caregiver burden explained most variance in family caregivers’ mental HRQoL. That is, the lower the perceived caregiver burden, the higher the mental HRQoL [in line with (26, 41)]. As described before, caregiver burden entails the demands, challenges, and stressors experienced by those who are providing care (41) on several dimensions, i.e., negative feelings resulting from informal caregiving, lack of support from family and friends, relationship problems, mental and physical health problems, problems with activities of daily living, and financial problems due to caregiving responsibilities (50). The present results indicate that the sum of these dimensions of caregiver burden explain variance in the mental (and in the bivariate analyses also in the physical) HRQoL, but it remains unclear whether certain dimensions clarify more/less variance in HRQoL compared to others, so that precise support approaches to reduce perceived caregiver burden cannot yet be defined. Interestingly, more objective indicators of caregiver burden (i.e., capacity of informal care) did not significantly predict the HRQoL (26), suggesting that it is not the actual care provided, but the individual’s perception of the care situation that has an impact on the HRQoL. This provides an initial benchmark for developing appropriate services, such as psychosocial interventions. Evidence is lacking, but first data on interventions for parents of autistic youth and adults seem promising, as mindfulness-based group interventions significantly reduced parental stress (71, 72). In addition to formal services, informal social support (i.e., support from unpaid sources such as family members, friends, or acquaintances) was found to reduce both subjective caregiver burden and mental HRQoL (41).

Better school education has previously been reported to increase maternal mental well-being (40). Interestingly, our data revealed a significant inverse association, indicating that higher school education predicted lower mental HRQoL. It is possible that family caregivers with higher school education tend to have, in turn, higher ambitions for their own lives and for the life of their care recipients, but are constrained by the demands of caregiving. Well-educated people were found to have higher levels of dissatisfaction, and mental distress was largely reduced by paid work (73), but employment is often negatively affected by caregiving demands among caregivers of autistic adults. Such associations need to be investigated in future research.

To our knowledge, this is the first study to show an association between care recipient’s age at ASD diagnosis and family caregiver’s mental HRQoL with an inverse relationship: Higher age at diagnosis predicted lower mental HRQoL. As mentioned in the Introduction, autistic adults face massive healthcare barriers, such as long waiting lists for diagnostics (3, 9, 10, 74). Delayed diagnosis might lead to delayed receipt of appropriate formal support. Such associations need further investigation, but it can be assumed that inadequate healthcare structures for autistic adults affect the whole family.

As expected by Sonido et al. (37), a higher amount of received formal services predicted lower mental HRQoL among family caregivers. The number of formal services could be a proxy for care recipients’ symptom severity and/or treatment needs. However, there was only a weak correlation between formal services and the severity of social interaction and communication symptoms. Recent studies have shown that the presence of care recipient’s depression (26) and general health deterioration (75) are associated with reduced caregiver well-being, but associations with HRQoL need to be clarified in future research. Furthermore, many family caregivers have had negative experiences with formal services (18), which may account for the negative association. In addition, in line with previous research, the presence of an ID (26) and higher caregiver’s age (26, 38) correlated with better mental HRQoL (only) in the bivariate analyses. Previous evidence confirmed that autistic adults without ID often report higher healthcare needs and barriers to accessing appropriate healthcare (76–78).

The remaining predictors were not found to significantly explain the variance of mental HRQoL in the current study, as was the relationship with the autistic adult. In contrast to Grootscholten et al. (24), the current study mainly included parental caregivers, which may result in a lack of variance to detect potential differences between different relationship groups (i.e., parents, siblings, spouses). Moreover, differences between maternal and paternal caregivers were not examined in the current study. Research on parents has shown that mothers often focus on caring for the autistic child and thus have a more intense bonding (79, 80). Nonetheless, in a recent meta-analysis, parental gender did not emerge as a significant moderator of proportions of levels of parental psychopathology, but the authors called for further investigation (81). Moreover, treatment-related costs were not associated with the family caregivers’ mental HRQoL even when family caregivers reported high amounts of care-related costs (35). This finding differed from qualitative findings (20), as almost all participating parental caregivers complained about the financial constraints they faced. Buescher et al. (82) also reported substantial costs for caregivers of autistic adults, such as medical services, employment support or accommodations, which can have a tremendous impact on families. Due to the high proportion of highly educated caregivers in the current study, the income of the sample is expected to be relatively high. In addition, the positive correlation between school education and treatment-related expenses showed that family caregivers with higher education reported more treatment-related expenses. Therefore, further research should examine the subjective financial burden.

Similar to mental HRQoL findings, subjective caregiver burden also correlated with the *physical HRQoL* with a small effect size, as caregiving may have negative long-term physical effects (e.g., fatigue, muscular tension, physiological exhaustion). Another correlation was found between the physical HRQoL and the severity of care-recipients’ ASD symptoms of repetitive, restrictive, and stereotype behaviors with an expected inverse relationship: The more severe the symptoms, the lower the physical HRQoL (22, 42). Managing the behavioral characteristics of care recipients may be a stressor for family caregivers, which is in accordance with research on caregivers of Alzheimer’s patients (83). In contrast, ASD symptom severity regarding specifics of social interactions and communication did not explain variance in this study [*cf.* (42)].

Nonetheless, the multiple linear regression model with *physical HRQoL* as the outcome was not statistically significant. The selection of predictors in this study was based on previous evidence that mainly focused on mental HRQoL without considering physical HRQoL, which could explain the lack of significance. Other variables that were not included in our analyses could explain more variance in family caregivers’ physical HRQoL, such as family caregivers’ mental and physical illness, social support, or coping strategies (26, 66).

### 4.3 Limitations and future directions

There are limitations that need to be considered in future research. First, because this was a cross-sectional study, no conclusions about causal relationships between variables and HRQoL can be drawn. Longitudinal studies would help to further describe the HRQoL of family caregivers of autistic adults over time in order to identify possible well-suited time points for different types of interventions to support caregivers. Second, a post-hoc analysis to attempt to estimate the power of the regression coefficients showed insufficient power (84). A sample size of approximately 250 relatives would have been required to achieve adequate power. Third, as no more recent SF-8 normative data from Germany exists, normative data collected in 2004 were used (59). Recent longitudinal data from the German general population showed that the overall HRQoL increased in the meantime (85). Taking this finding into account, the difference between the normative sample and the caregiver sample might be even larger, but further investigations are needed. In addition, study population and normative population were not stratified, for example, by age or gender, because these data were not available from the normative sample. Fourth, the generalizability of the data was limited by the characteristics of the sample. For example, the majority of the current sample were mothers with high levels of education and professional degree. Maternal perspectives are important but may not necessarily reflect those of other family members (8). Equal proportions of family members are needed, to examine possible differences in terms of different relationships with the autistic adult (e.g., spouses, siblings, children). Finally, family caregivers rated their relative’s symptom severity using self-developed items without prior psychometric validation.

## 5 Conclusion and implications

Family caregivers of autistic adults represent a highly under-recognized population in clinical research, healthcare, society, and policy. To our knowledge, this is the first study to comprehensively assess the physical and mental HRQoL of this population in Germany, not only by comparing the HRQoL of family caregivers with the general population, but also by identifying potential predictors. The results outlined considerably lower physical and mental HRQoL compared to the general population, emphasizing the need for action to improve family caregivers’ HRQoL, as well as certain implications for future research and healthcare. Especially the subjective burden of care was found to have impact on both dimensions of HRQoL in this study and seems to be a promising candidate for interventions. In addition to the few emerging studies on family caregivers’ mental HRQoL, the current findings reveal that physical HRQoL might depend on other predictors as mental HRQoL. There is a lack of research investigating this aspect: What causes the lower physical HRQoL? Moreover, healthcare providers working with autistic adults and their families should be aware of the potential impairment of caregivers’ health status and form a routine to assess and monitor caregivers’ HRQoL, for example as part of the intake assessment or in primary care (86). Further longitudinal research assessing diverse samples of family caregivers of autistic adults is needed, to elucidate specific underlying mechanisms and potential risk factors for HRQoL in this heterogeneous, complex population (9, 26, 45).

## Data availability statement

The raw data supporting the conclusions of this article will be made available by the authors, without undue reservation.

## Ethics statement

The studies involving humans were approved by Local Psychological Ethics Committee at the Center for Psychosocial Medicine at the University Medical Center Hamburg-Eppendorf. The studies were conducted in accordance with the local legislation and institutional requirements. The participants provided their written informed consent to participate in this study.

## Author contributions

SD: Conceptualization, Data curation, Formal analysis, Investigation, Supervision, Visualization, Writing – original draft, Writing – review & editing. SB: Formal analysis, Writing – original draft, Writing – review & editing. PG: Investigation, Writing – original draft, Writing – review & editing. HK: Conceptualization, Investigation, Writing – original draft, Writing – review & editing. DS: Conceptualization, Funding acquisition, Supervision, Writing – original draft, Writing – review & editing. AK: Conceptualization, Funding acquisition, Supervision, Writing – original draft, Writing – review & editing. PR: Investigation, Writing – original draft, Writing – review & editing. FE: Writing – original draft, Writing – review & editing, Investigation. KV: Conceptualization, Funding acquisition, Supervision, Writing – original draft, Writing – review & editing. HS: Conceptualization, Data curation, Funding acquisition, Investigation, Project administration, Writing – original draft, Writing – review & editing, Supervision, Validation. ND: Conceptualization, Funding acquisition, Investigation, Project administration, Writing – original draft, Writing – review & editing. JP: Conceptualization, Formal analysis, Funding acquisition, Investigation, Project administration, Supervision, Visualization, Writing – original draft, Writing – review & editing.

## Footnotes

^1^ https://osf.io/5x8pg

## Abbreviations

ASD: Autism Spectrum Disorder
CarerQoL-7D: Care-Related Quality of Life of Informal Caregivers
DSM-5: Diagnostic and Statistical Manual of Mental Disorders
GDW: General Dominance Weights
HRQoL: Health-related Quality of Life
ID: Intellectual Disability
MCS: Mental Component Summary Score
PCS: Physical Component Summary Score
QoL: Quality of Life
SF-8: Short-Form Health Survey
VIF: Variance Inflation Factor
